# Intrinsic mechanisms underlying urban–rural disparities in physical activity and their changes in China: evidence from China General Social Survey during 2010–2021

**DOI:** 10.3389/fpubh.2026.1782057

**Published:** 2026-04-17

**Authors:** Chengcheng Huang, Shanjun Bao

**Affiliations:** 1 College of Science and Technology, Wuhan Sports University, Wuhan, China; 2 School of Sports Training, Wuhan Sports University, Wuhan, China

**Keywords:** CGSS data, intrinsic mechanisms, physical activity, temporal change, urban and rural residents

## Abstract

With rapid socioeconomic development in China, significant disparities have emerged between urban and rural residents exhibit in terms of participation, frequency, and forms of physical activity. These differences reflect not only the uneven distribution of economic, cultural, and social resources between urban and rural areas, but also variations in health literacy and lifestyles across different social groups. Based on data from the China General Social Survey (CGSS) for 2010 and 2021, this study employs a multidimensional regression analysis model to compare differences in physical exercise between urban and rural residents in China at these two time points. It also explores in depth the multiple factors underlying these differences and their interactions. The results indicate that urban residents consistently engaged in physical activity more frequently than rural residents, although the frequency of physical activity for both urban and rural residents increased from 2010 to 2021. Income level, years of education, frequency of social interaction, and internet use are all significantly associated with physical activity frequency, with varying trends across time and region. The positive effects of education and internet use gradually strengthened in rural areas, while the influence of social interaction frequency weakened in urban areas. It is recommended that public health policies pay greater attention to rural areas to promote balanced development of physical activity between urban and rural residents, thereby narrowing the urban–rural gap and contributing to the national fitness campaign and the realization of a Healthy China.

## 1 Introduction

Disparities in physical activity between urban and rural residents have long been a topic of significant interest in the fields of sociology, public health, and exercise science. With the rapid socioeconomic development in China, urban and rural residents show marked differences in physical activity participation, frequency, and forms (1–3). These disparities not only reflect the uneven distribution of economic, cultural, and social resources between urban and rural areas, but also reveal variations in health literacy and lifestyles among different social groups (4, 5). A deeper understanding of these disparities is crucial for advancing the national fitness agenda and achieving the goal of building a Healthy China.

In recent years, alongside rapid economic development and accelerated urbanization, the lifestyles and health status of urban and rural residents in China have undergone substantial changes (6, 7). Urban residents, benefiting from better economic conditions, higher educational attainment, and more abundant social resources, are more inclined to engage in various forms of physical activity (3, 8). Conversely, rural residents generally show lower levels of participation and frequency in physical activity, partly due to relatively poorer economic conditions, lower education levels, and limited social resources (9). Such urban–rural disparities not only affects residents’ physical health, but also have profound implications for social harmony and development.

Based on data from the China General Social Survey (CGSS) conducted in 2010 and 2021, this study employs a multidimensional regression analysis model to compare differences in physical exercise between urban and rural residents in China at these two time points. It also explores in depth the multiple factors underlying these differences and their interrelationships. Previous studies have examined the reasons behind these differences from economic, social, and cultural perspectives (10–13). However, most of these studies are limited to a single time point and lack a systematic analysis of changes over time. Moreover, existing studies often exhibit shortcomings in variable selection and analytical methods, thus failing to fully unravel the complex mechanisms underlying urban–rural disparities (14, 15). As a nationally representative and comprehensive social survey, the CGSS provides rich socioeconomic, cultural, and health-related data, forming a solid foundation for the present study. Accordingly, this study addressed the following research questions: (1) What are the specific trends in urban–rural disparities in physical activity between 2010 and 2021? (2) Have the effects of factors such as income level, years of education, social interaction frequency, and internet use on urban–rural disparities in physical activity changed significantly over time? (3) How can policy interventions effectively reduce these disparities? Analyzing CGSS data from 2010 and 2021 will help to comprehensively understand the internal mechanisms of physical activity and dynamic changes of urban–rural disparities in physical activity, thereby providing a scientific basis for formulating more targeted public health policies.

## 2 Methods

### 2.1 Data sources

The China General Social Survey (CGSS) is a nationwide, longitudinal social survey project conducted by the China Survey and Data Center at Renmin University of China. It employs a multistage stratified probability sampling method, covering all 31 provinces, autonomous regions, and municipalities directly under the central government. The survey targets Chinese citizens aged 18 and older, and the data collected is made publicly available (16). The CGSS encompasses multiple dimensions, including individual demographic characteristics, socioeconomic status, health behaviors, and social attitudes. It is distinguished by strong national representativeness and cross-year comparability. All survey procedures and methods comply with relevant ethical guidelines and regulations, and informed consent has been obtained from all participants.

#### 2.1.1 Statistical analysis of sample data

**Figure 1:**
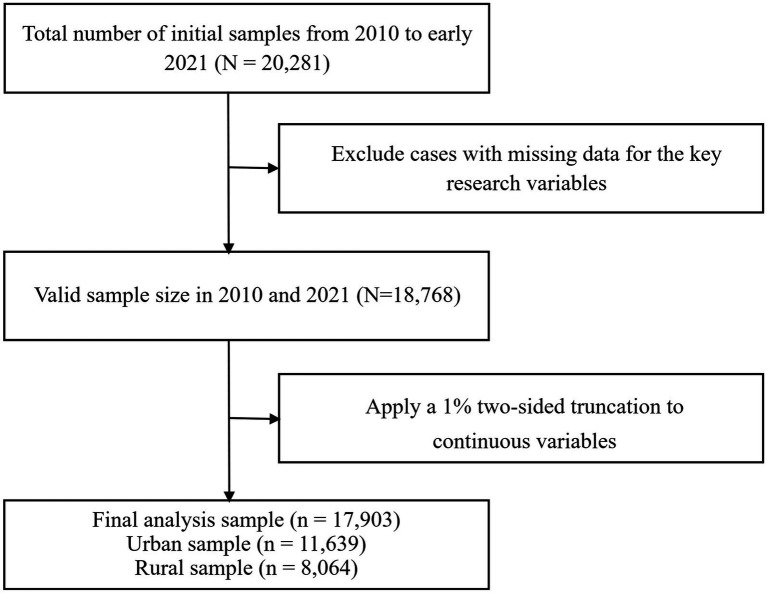
Data filtering flowchart.

This study selected data from two time periods: 2010 and 2021. First, 2010 marked a critical phase in China’s efforts to build a moderately prosperous society in all respects, while 2021 was a milestone year marking the full realization of this goal. The evolution of the data fully reflects the changing trends in urban–rural disparities in physical activity during China’s period of development and transformation. Second, the two datasets maintain consistency in the measurement methods for core variables, providing a solid foundation for longitudinal comparisons. Third, the 2021 data represent the most recently released version of this survey, ensuring the timeliness of the study. The data processing workflow is shown in Figure 1. The initial sample sizes for the 2010 and 2021 surveys were 11,783 and 8,498, respectively. We performed the following data cleaning steps to construct the final analytical sample: (1) retained respondents with no missing values for core variables (physical activity frequency, household registration, income, education, etc.); and (2) excluded outliers—specifically, values in the top and bottom 1% of the distribution for key continuous variables (such as income)—to mitigate the impact of anomalies. Following these procedures, a total of 11,639 valid samples were obtained for analysis, comprising 7,097 samples from 2010 and 4,542 samples from 2021.

#### 2.1.2 Demographic statistics

In terms of urban–rural distribution, there were 11,639 urban samples (59.07%) and 8,064 rural samples (40.93%). Regarding regional distribution, 7,877 samples (43.99%) were from the eastern region, 5,645 samples (31.53%) from the central region, and 4,381 samples (24.47%) from the western region. Concerning age structure, there were 3,212 samples (17.94%) in the 18–30 age group, 5,843 samples (32.64%) in the 31–45 age group, 5,678 respondents (31.71%) aged 46–60, and 3,170 respondents (17.71%) aged 61 and older. By gender, there were 8,493 male respondents (47.44%) and 9,410 female respondents (52.56%). Regarding occupational distribution, there were 4,832 manual laborers (26.99%), 4,156 non-manual workers (23.22%), and 8,915 unemployed individuals (including retirees and students) (49.79%). The demographic characteristics of the sample generally align with the national profile, providing a reliable basis for urban–rural comparative analysis.

### 2.2 Variable selection

#### 2.2.1 Dependent variable

The dependent variable in this study is the frequency of physical activity among urban and rural residents. This variable is derived from responses to the CGSS question, “How often do you usually engage in physical exercise?” Physical activity frequency is measured on a 5-point scale, with higher values indicating a greater exercise frequency. Specifically, the scale is defined as follows: 1 = never exercise, 2 = occasionally exercise, 3 = 1-2 times per month, 4 = 1-2 times per week, and 5 = exercise daily or almost daily. This variable reflects respondents’ physical activity habits over time and provides a reasonably accurate measure of their actual physical activity levels.

#### 2.2.2 Independent variables

Based on existing literature and theoretical framework, this study selected independent variables such as income level, years of education, social interactions, and internet use to explore the intrinsic mechanisms influencing physical activity frequency among urban and rural residents (17). Income level is an important economic factor affecting physical activity. This study measured it as the natural logarithm of the respondents’ total annual income plus one. Years of education reflects the respondents’ educational attainment, which influences health awareness and knowledge about physical activity. Educational attainment is converted into a ranked variable ranging from 0 to 18 years based on the following mapping: no schooling = 0, elementary school = 6, junior high school = 9, high school = 12, junior college/technical school = 13, college = 15, undergraduate = 16, and postgraduate = 18. Frequency of social interaction reflects the respondents’ social network and support system. It is measured on a 5-point scale (1–5), with higher values indicating more frequent social interactions. Frequency of internet use reflects the convenience and diversity of access to health information. It is also measured on a 5-point scale (1–5), with higher values indicating more frequent internet use.

#### 2.2.3 Control variables

To accurately estimate the net effects of the core independent variables (income, education, social engagement, and internet use) on the frequency of physical activity, and to control for confounding factors stemming from demographic differences in urban–rural comparisons, this study incorporated multilevel control variables into the model. The selection of these variables was guided by the social-ecological model of health behavior and relevant literature, aiming to account for potential relevant factors at the individual, household, and societal levels.

(1) Individual demographic characteristics: Gender is included to account for known differences in physical activity participation between males and females. Age and its squared term (age^2^) are incorporated to capture the nonlinear patterns of physical activity across the life course. (2) Family factors: Marital status and the presence of children are included to account for the impact of family responsibilities and structure on individuals’ discretionary time and lifestyle. This is particularly important given the significant differences in family patterns between urban and rural areas. (3) Health status: Self-rated health serves as a key control variable. Health status is both associated with physical activity and a potential correlate of participation. Including it in the model helps partially address reverse causality, allowing for a clearer identification of the association between socioeconomic factors and behavior. (4) Socioeconomic status and regional context: To address the vast developmental disparities across China’s extensive territory, this study controls for these factors as follows. First, occupational type (job1, job2, job3): Occupations are categorized as “unemployed” (including students, retirees, and the jobless, set as the reference group), “manual labor,” and “non-manual labor.” (18) This classification effectively controls for potential confounding effects of the physical exertion inherent in occupations on the measurement of “physical exercise” as leisure-time physical activity. For example, farmers’ daily manual labor may substitute for dedicated physical exercise. Second, household registration (urban): In some models, this serves as a core explanatory variable; in others, it functions as a control variable or the basis for interaction terms. It inherently captures the fundamental structural differences between urban and rural areas.

The inclusion of these control variables enables a more precise analysis of the underlying mechanisms and historical changes in physical exercise disparities between urban and rural residents. The specific selection of each variable is detailed in Table 1.

**Table 1:** Variable definitions and measurements.

Variable type	Symbol	Variable name	Description
Dependent	exec	Physical activity	Frequency of physical activity (1–5); higher values indicating higher frequency.
Independent	lninc	Income level	Natural logarithm of (total annual income + 1)
	edu	Years of education	0–18 years, based on educational attainment mapping
	social	Social interaction	Social interaction frequency (1–5)
	nett	Internet use	Internet accessing frequency (1–5)
Control	gender	Gender	Male = 1, Female = 0
	age	Age	Interview year–Birth year
	age^2^	Age squared	(Age × Age)/100
	marriage	Marital status	Married = 1, Not married = 0
	child	Presence of children	Has children = 1, No children = 0
	health	Health status	Self-rated health (1 = very poor to 5 = very good)
	job1	Not employed	Yes = 1, No = 0
	job2	Manual labor	Yes = 1, No = 0
	job3	Non-manual labor	Yes = 1, No = 0

#### 2.2.4 Model specification

To examine the variability in physical activity frequency across different years, this study establishes Empirical Model 1:


$${\text{exec}}_{\mathrm{it}}=\beta_{0}+\beta_{1}{\text{year}}_{\mathrm{it}}+\beta_{n}{\text{controls}}_{\mathrm{it}}+\varepsilon_{\mathrm{it}}$$ (1)

To explore the differences in physical activity frequency between urban and rural residents, Empirical Model 2 is employed:


$${\text{exec}}_{\mathrm{it}}=\beta_{0}+\beta_{1}{\text{urban}}_{\mathrm{it}}+\beta_{2}{\text{year}}_{\mathrm{it}}+\beta_{n}{\text{controls}}_{\mathrm{it}}+\varepsilon_{\mathrm{it}}$$ (2)

To investigate the effects of income level, years of education, social interaction frequency, and internet use frequency on physical activity frequency, Empirical Model 3 is specified:


$$\begin{array}{l} {\text{exec}}_{\mathrm{it}}=\beta_{0}+\beta_{1}{\text{lninc}}_{\mathrm{it}}+\beta_{2}{\mathrm{edu}}_{\mathrm{it}}+\beta_{3}{\text{social}}_{\mathrm{it}}+\beta_{4}{\text{nett}}_{\mathrm{it}}\\ +\beta_{5}{\text{urban}}_{\mathrm{it}}+\beta_{6}{\text{year}}_{\mathrm{it}}+\beta_{n}{\text{controls}}_{\mathrm{it}}+\varepsilon_{\mathrm{it}} \end{array}$$ (3)

To examine the differential effects of these factors on urban and rural residents, Empirical Model 4 includes interaction terms:


$$\begin{array}{l} {\text{exec}}_{\mathrm{it}}=\beta_{0}+\beta_{1}{\text{lninc}}_{\mathrm{it}}+\beta_{2}{\text{lninc}}_{\mathrm{it}}*{\text{urban}}_{\mathrm{it}}+\beta_{3}{\mathrm{edu}}_{\mathrm{it}}\\ +\beta_{4}{\mathrm{edu}}_{\mathrm{it}}*{\text{urban}}_{\mathrm{it}}+\beta_{5}{\text{social}}_{\mathrm{it}}+\beta_{6}{\text{social}}_{\mathrm{it}}*{\text{urban}}_{\mathrm{it}}\\ +\beta_{7}{\text{nett}}_{\mathrm{it}}+\beta_{8}{\text{nett}}_{\mathrm{it}}*{\text{urban}}_{\mathrm{it}}+\beta_{9}{\text{urban}}_{\mathrm{it}}+\beta_{10}{\text{year}}_{\mathrm{it}}\\ +\beta_{n}{\text{controls}}_{\mathrm{it}}+\varepsilon_{\mathrm{it}} \end{array}$$ (4)

To investigate the mediating role of health awareness in the relationship between education and physical activity, a mediation model is constructed (Equations 5–7).


$${\text{exec}}_{\mathrm{it}}=\beta_{0}+\beta_{1}{\mathrm{edu}}_{\mathrm{it}}+\beta_{n}{\text{controls}}_{\mathrm{it}}+\varepsilon_{\mathrm{it}}$$ (5)

$${\text{health}}_{\mathrm{it}}=\beta_{0}+\beta_{1}{\mathrm{edu}}_{\mathrm{it}}+\beta_{n}{\text{controls}}_{\mathrm{it}}+\varepsilon_{\mathrm{it}}$$ (6)

$${\text{exec}}_{\mathrm{it}}=\beta_{0}+\beta_{1}{\mathrm{edu}}_{\mathrm{it}}+\beta_{2}{\text{health}}_{\mathrm{it}}+\beta_{n}{\text{controls}}_{\mathrm{it}}+\varepsilon_{\mathrm{it}}$$ (7)

Similar mediation models are specified for social interaction (via social participation opportunities (Equations 8–10), and internet use (via access to information resources, Equations 11–13). In these equations, “*i*” denotes individuals, “*t*” denotes the years (2010 or 2021), controls*_it* represents the set of control variables, $\beta_{0}$ is the intercept term, $\beta_{1}$ is the coefficient of the core explanatory variable, and $\varepsilon_{\mathrm{it}}$ denotes the random error term.


$${\text{exec}}_{\mathrm{it}}=\beta_{0}+\beta_{1}{\text{social}}_{\mathrm{it}}+\beta_{n}{\text{controls}}_{\mathrm{it}}+\varepsilon_{\mathrm{it}}$$ (8)

$${\text{cysocial}}_{\mathrm{it}}=\beta_{0}+\beta_{1}{\text{social}}_{\mathrm{it}}+\beta_{n}{\text{controls}}_{\mathrm{it}}+\varepsilon_{\mathrm{it}}$$ (9)

$${\text{exec}}_{\mathrm{it}}=\beta_{0}+\beta_{1}{\text{social}}_{\mathrm{it}}+\beta_{2}{\text{cysocial}}_{\mathrm{it}}+\beta_{n}{\text{controls}}_{\mathrm{it}}+\varepsilon_{\mathrm{it}}$$ (10)

$${\text{exec}}_{\mathrm{it}}=\beta_{0}+\beta_{1}{\text{nett}}_{\mathrm{it}}+\beta_{n}{\text{controls}}_{\mathrm{it}}+\varepsilon_{\mathrm{it}}$$ (11)

$${\text{medd}}_{\mathrm{it}}=\beta_{0}+\beta_{1}{\text{nett}}_{\mathrm{it}}+\beta_{n}{\text{controls}}_{\mathrm{it}}+\varepsilon_{\mathrm{it}}$$ (12)

$${\text{exec}}_{\mathrm{it}}=\beta_{0}+\beta_{1}{\text{nett}}_{\mathrm{it}}+\beta_{2}{\text{medd}}_{\mathrm{it}}+\beta_{n}{\text{controls}}_{\mathrm{it}}+\varepsilon_{\mathrm{it}}$$ (13)

#### 2.2.5 Sub-sample descriptive statistics

Table 2 presents the descriptive statistics for urban and rural sub-samples, showing the means and standard deviations for core variables including physical activity frequency, income level, years of education, social interaction frequency, and internet use frequency. Statistical analysis reveals that the mean physical activity frequency for urban residents (mean = 2.766) is significantly higher than that of rural residents (1.886), with standard deviations of 1.556 and 1.406, respectively, indicating that urban residents are more active physically. Regarding income level, the mean income for urban residents is 9.838, significantly higher than the 8.476 for rural residents, with standard deviations of 3.946 and 4.142, respectively, suggesting a considerable income gap. In terms of education, urban residents have an average of 10.098 years of schooling, significantly higher than the 6.351 years for rural residents, with a wider distribution in urban residents. Urban residents also report higher frequencies of social interaction (mean = 2.712 vs. 2.521) and internet use (mean = 3.042 vs. 2.094), reflecting the greater social interaction engagement and internet penetration in urban areas. Control variables such as gender, age, marital status, presence of children, health status, and occupation type also shown significant differences between urban and rural areas, as detailed in Table 2.

**Table 2:** Descriptive statistical analysis.

Variable	Symbol	Urban areas			Rural areas		
		Mean	Standard deviation	Sample size	Mean	Standard deviation	Sample size
Physical activity	exec	2.766	1.556	11,639	1.886	1.406	8,064
Income level	lninc	9.838	3.946	11,639	8.476	4.142	8,064
Years of education	edu	10.098	4.517	11,639	6.351	3.852	8,064
Social interaction	social	2.712	1.048	11,639	2.521	1.071	8,064
Internet use	nett	3.042	1.841	11,639	2.094	1.679	8,064
Gender	gender	0.462	0.499	11,639	0.481	0.500	8,064
Age	age	47.637	16.943	11,639	51.058	15.879	8,064
Age squared	age2	25.564	17.032	11,639	28.590	16.236	8,064
Marital status	marriage	0.743	0.437	11,639	0.799	0.401	8,064
Children	child	0.830	0.376	11,639	0.909	0.288	8,064
Health status	health	3.670	1.035	11,639	3.406	1.189	8,064
Not employed	job1	0.487	0.500	11,639	0.484	0.500	8,064
Manual labor	job2	0.167	0.373	11,639	0.429	0.495	8,064
Non-manual labor	job3	0.346	0.476	11,639	0.086	0.281	8,064

#### 2.2.6 Controlling for multiple comparisons

This study involved testing multiple sets of interaction terms in regression analyses, as well as conducting subgroup analyses based on urban/rural status and year. Instead of applying correction methods such as the Bonferroni adjustment, we comprehensively evaluated the robustness of the findings by clearly specifying the nature of the analyses, presenting full confidence intervals, and emphasizing the interpretation of effect sizes. First, the interaction term analysis in this study (Model 4) was guided by a pre-specified theoretical hypothesis: that the associations of income, education, social connections, and internet use with the frequency of physical exercise may differ between urban and rural areas. These interaction tests represent confirmatory analyses; the number of tests is limited, and they are explicitly theory-driven. We did not apply multiple comparison corrections to *p*-values to avoid increasing the risk of Type II errors due to excessive conservatism. Second, the subgroup analyses in this study (by urban/rural status and year) are considered exploratory and are intended to generate research hypotheses rather than to confirm them.

## 3 Results

### 3.1 Sample characteristics

To evaluate the potential impact of changes in sample composition on the estimates of the “year” variable, this study conducted a direct comparison and statistical tests of key demographic characteristics between the 2010 and 2021 analytical samples. Table 3 presents the differences in distribution, test statistics, and significance levels for the two samples across urban–rural distribution, age structure, educational attainment, occupational type, and marital status.

**Table 3:** Comparison of sample characteristics between 2010 and 2021.

Variable	2010 sample (*N* = 7,097)	2021 sample (*N* = 4,542)	Test statistic	*p*-value
Proportion of urban registered residence (%)	59.4	67.8	*χ*^2^ = 84.28	<0.001
Age (mean ± SD, years)	47.2 ± 16.8	51.2 ± 15.9	*t* = −12.89	<0.001
Proportion of population aged 60 and above (%)	21.5	30.1	*χ*^2^ = 110.56	<0.001
Years of education (mean ± SD, years)	8.1 ± 4.2	9.3 ± 4.1	*t* = −14.21	<0.001
Proportion of individuals with a college degree or above (%)	18.7	29.4	*χ*^2^ = 187.34	<0.001
Proportion of non-agricultural occupations (%)	52.9	55.8	*χ*^2^ = 9.45	0.002
Marriage rate (%)	81.2	70.5	*χ*^2^ = 175.83	<0.001

SD stands for standard deviation. The inter-group difference is calculated by subtracting the 2010 value from the 2021 value. The testing methods used are the independent samples *t*-test for continuous variables and the chi-square test for categorical variables.

The results in Table 3 indicate that the two analytical samples from 2010 and 2021 exhibit significant differences in several key characteristics, specifically:

(1) Changes in Urban–Rural Structure: In the 2021 sample, the proportion of the urban registered population increased by 8.4 percentage points compared to 2010 (*χ*^2^ = 84.28, *p* < 0.001). Data from the study sample indicate that the mean frequency of physical exercise among urban residents was 2.77, significantly higher than the 1.89 reported for rural residents (*t* = 42.51, *p* < 0.001). Therefore, if urban–rural differences are not adequately controlled for in the model, the coefficient of the “Year” variable may partially capture this structural change, leading to an overestimation of the net increase in physical exercise frequency.
(2) Population Aging Trends: The average age of the 2021 sample increased significantly by 4.0 years compared to 2010 (*t* = −12.89, *p* < 0.001), and the proportion of the population aged 60 and older rose from 21.5 to 30.1% (*χ*^2^ = 110.56, *p* < 0.001). The sample data indicate a nonlinear relationship between exercise frequency and age; the average exercise frequency for the 60-and-older group was 2.01, significantly lower than that of other age groups (*F* = 156.34, *p* < 0.001). Increasing population aging may exert a downward influence on overall exercise frequency. If the model does not adequately control for the nonlinear effect of age, the “year” variable may underestimate the true effectiveness of policies promoting physical exercise.
(3) Human capital has increased significantly: The average years of education in the 2021 sample increased by 1.2 years compared to 2010 (*t* = −14.21, *p* < 0.001), and the proportion of individuals with an associate’s degree or higher rose from 18.7 to 29.4% (*χ*^2^ = 187.34, *p* < 0.001). The sample data show that years of education are significantly positively correlated with the frequency of physical exercise (correlation coefficient *r* = 0.31, *p* < 0.001). This improvement in human capital may be partially captured by the “year” variable, potentially introducing an upward bias in estimating the time effect.
(4) Changes in occupational structure and marital status: In the 2021 sample, the proportion of individuals employed in non-agricultural occupations increased by 2.9 percentage points compared to 2010 (*χ*^2^ = 9.45, *p* = 0.002), while the proportion of married individuals decreased significantly by 10.7 percentage points (*χ*^2^ = 175.83, *p* < 0.001). Occupational type is closely related to access to resources for physical exercise, whereas marital status influences the allocation of time for family responsibilities. These changes may both have important implications for the frequency of physical exercise.

Based on the analysis above, the two sample cohorts from 2010 and 2021 exhibit significant differences in urban–rural composition, age structure, educational attainment, occupational type, and marital status. Directly comparing the two samples without controlling for compositional effects could lead to biased estimates of the “year” variable: rising urbanization rates and increased human capital may cause an overestimation of the time effect, while an aging population may cause an underestimation. To minimize the impact of changes in sample composition on the time effect, this study includes control variables such as age, age squared, years of education, urban/rural household registration, occupational type, and marital status in the regression model. The goal is to statistically reduce the compositional effect, thereby producing an estimate of the “year” coefficient that more accurately reflects the true period effect.

### 3.2 Changes in physical activity between urban and rural residents

Table 4 presented physical activity levels of urban and rural residents in 2010 and 2021. In 2010, the mean physical activity frequency was 2.511 for urban residents and 1.471 for rural residents, with a significant difference at the 1% level (*t* = 40.529). Similarly, in 2021, urban residents reported a mean frequency of 3.164, compared to 2.416 for rural residents, a difference also significant at 1% level (*t* = 21.182). Moreover, comparing 2010 and 2021 data reveals that physical activity frequency increased for urban and rural residents over this period, suggesting a growing willingness and behavior toward physical activity among Chinese residents and reflecting the positive impact of national fitness policies and the Healthy China initiative.

**Table 4:** Physical activity and key variables: urban vs. rural residents (2010 and 2021).

Variable	Symbol	2010			2021		
		Urban	Rural	*T*-stat	Urban	Rural	*T*-stat
Physical activity	exec	2.511	1.471	40.529***	3.164	2.416	21.182***
Income level	lninc	9.862	8.567	17.729***	9.801	8.359	15.027***
Years of education	edu	9.874	5.757	51.726***	10.449	7.108	34.677***
Social interaction	social	2.759	2.432	17.112***	2.637	2.635	0.088
Internet use	nett	2.482	1.323	41.260***	3.915	3.078	21.225***
Gender	gender	0.475	0.492	−1.824*	0.441	0.466	−2.224**
Age	age	46.391	48.659	−7.630***	49.586	54.117	−11.608***
Age squared	age2	24.078	25.931	−6.258***	27.884	31.981	−10.238***
Marital status	marriage	0.781	0.840	−7.858***	0.683	0.747	−6.279***
Children	child	0.847	0.927	−13.002***	0.803	0.885	−10.030***
Health status	health	3.706	3.478	10.776***	3.614	3.313	12.395***
Not employed	job1	0.437	0.239	22.201***	0.565	0.798	−22.754***
Manual labor	job2	0.209	0.690	−58.962***	0.100	0.096	0.635
Non-manual labor	job3	0.354	0.071	36.564***	0.335	0.106	24.999***

*, ** and *** represent 10%, 5% and 1% significance levels.

### 3.3 Regression analysis and interaction model

Table 5 presents the regression results for the baseline models. Column (1) shows that the coefficient for the year variable is 0.047, significant at the 1% level, indicating a consistent upward trend in physical activity frequency from 2010 to 2021. Column (2) reveals that the coefficient for the urban dummy variable is 0.736, also significant at the 1% level, confirming that urban residents engage in physical activity more frequently than rural residents.

**Table 5:** Regression analysis for baseline models.

Variable	(1) exec	(2) exec	(3) exec	(4) exec
year	0.047^***^	0.056^***^	0.032^***^	0.034^***^
	(0.002)	(0.002)	(0.002)	(0.002)
urban		0.736^***^	0.392^***^	1.218^***^
		(0.022)	(0.022)	(0.061)
lninc			0.030^***^	0.021^***^
			(0.002)	(0.003)
edu			0.094^***^	0.037^***^
			(0.003)	(0.003)
social			0.148^***^	0.056^***^
			(0.009)	(0.013)
net			0.105^***^	0.017^*^
			(0.008)	(0.010)
urban × lninc				0.018^***^
				(0.005)
urban × edu				0.106^***^
				(0.004)
urban × social				0.183^***^
				(0.019)
urban × nett				0.137^***^
				(0.013)
gender	0.211^***^	0.211^***^	0.041^**^	0.037^*^
	(0.021)	(0.021)	(0.020)	(0.019)
Age	0.023^***^	0.023^***^	0.034^***^	0.027^***^
	(0.004)	(0.004)	(0.004)	(0.004)
age^2^	−0.017^***^	−0.018^***^	−0.016^***^	−0.012^***^
	(0.004)	(0.004)	(0.004)	(0.004)
marriage	0.016	0.027	−0.023	−0.003
	(0.032)	(0.031)	(0.029)	(0.028)
child	−0.488^***^	−0.442^***^	−0.195^***^	−0.093^**^
	(0.046)	(0.045)	(0.043)	(0.041)
health	0.170^***^	0.145^***^	0.082^***^	0.093^***^
	(0.010)	(0.010)	(0.010)	(0.009)
job1	0.726^***^	0.510^***^	0.382^***^	0.465^***^
	(0.029)	(0.029)	(0.027)	(0.027)
job3	0.871^***^	0.497^***^	0.056^*^	0.052^*^
	(0.030)	(0.031)	(0.031)	(0.030)
_cons	−92.997^***^	−111.400^***^	−64.715^***^	−67.960^***^
	(4.147)	(4.075)	(4.449)	(4.271)
*N*	19,703	19,703	19,703	19,703
*R* ^2^	0.125	0.171	0.261	0.324

^*^, ^**^ and ^***^ represent 10%, 5%, and 1% significance levels.

Column (3) demonstrates income level, years of education, social interaction frequency, and internet use frequency all have significant associations with physical activity frequency, with coefficients of 0.030, 0.094, 0.148, and 0.105, respectively. Column (4) introduces interaction terms between the urban dummy and the four key independent variables. The positive and significant coefficients for these interaction terms (urban × linic = 0.018, urban × edu = 0.106, urban × social = 0.0183, *p* = 0.018) suggesting the positive effects of income, education, social interaction, and internet use on physical activity are stronger for urban residents than for rural residents.

### 3.4 Mediation effect and mechanism analysis

This section utilizes a mediation model to investigate the underlying mechanisms driving differences in physical exercise between urban and rural residents. Specifically, we examine the pathways through which educational attainment affects physical exercise frequency via health awareness; social interaction frequency influences physical exercise frequency through opportunities for social participation; and internet use impacts physical exercise frequency by providing access to information resources (19). The study employs the bootstrap method with 500 resamples to test for mediation effects and to calculate the 95% confidence interval (95% CI) for the indirect effect. If the confidence interval does not include zero, the mediation effect is considered significant. Table 6 presents the results of the regression analysis and bootstrap tests for the mediation effects.

(1) Educational Level, Health Awareness, and Physical Exercise: The results in Column (1) show that the total effect of educational level on the frequency of physical exercise is 0.137 (*p* < 0.01). Column (2) indicates that educational level has a significant positive effect on health awareness (coefficient = 0.031, *p* < 0.01). In Column (3), after controlling for health awareness, the direct effect of educational level is 0.134 (*p* < 0.01), and the effect of health awareness is 0.115 (*p* < 0.01). Bootstrap test results show that the indirect effect of educational level on physical exercise via health awareness is 0.003, with a 95% confidence interval of [0.002, 0.005], which does not include zero, indicating that the mediating effect of health awareness is significant. This suggests a link between educational attainment, health awareness and participation in physical exercise.
(2) Social Frequency, Opportunities for Social Participation, and Physical Exercise: Column (4) shows that the overall effect of social frequency on physical exercise is 0.211 (*p* < 0.01). Column (5) indicates that social frequency has a significant positive effect on opportunities for social participation (coefficient = 0.680, *p* < 0.01). Column (6) shows that, after controlling for opportunities for social participation, the direct effect of social frequency is 0.085 (*p* < 0.01), and the effect of opportunities for social participation is 0.186 (*p* < 0.01). Bootstrap test results reveal that the indirect effect is 0.127, with a 95% confidence interval of [0.115, 0.140], which does not include zero, indicating that the mediating effect of opportunities for social participation is significant. These findings suggest a correlation between social frequency and opportunities for social participation and physical activity among individuals.
(3) Internet Use, Access to Information Resources, and Physical Exercise. Column (7) shows that the total effect of internet use on physical exercise is 0.259 (*p* < 0.01). Column (8) indicates that internet use has a significant positive effect on access to information resources (coefficient = 1.029, *p* < 0.01). In Column (9), after controlling for access to information resources, the direct effect of internet use is 0.162 (*p* < 0.01), and the effect of access to information resources is 0.094 (*p* < 0.01). Bootstrap test results reveal an indirect effect of 0.097, with a 95% confidence interval of [0.090, 0.104], which does not include zero, indicating that the mediating effect of access to information resources is significant. This suggests that there are clear correlations and underlying explanations for the relationship between internet use, residents’ access to information and resources about physical activity, and how often people exercise.

**Table 6:** Regression results of mediation effects.

Variable	(1) exec	(2) health	(3) exec	(4) exec	(5) cysocial	(6) exec	(7) exec	(8) medd	(9) exec
edu	0.137^***^	0.031^***^	0.134^***^						
	(0.003)	(0.002)	(0.003)						
social				0.211^***^	0.680^***^	0.085^***^			
				(0.010)	(0.019)	(0.010)			
cysocial						0.186^***^			
						(0.003)			
nett							0.259^***^	1.029^***^	0.162^***^
							(0.007)	(0.016)	(0.007)
medd									0.094^***^
									(0.003)
gender	0.068^***^	0.106^***^	0.055^***^	0.197^***^	−0.003	0.198^***^	0.170^***^	0.839^***^	0.091^***^
	(0.021)	(0.015)	(0.021)	(0.022)	(0.042)	(0.020)	(0.021)	(0.050)	(0.021)
age	0.029^***^	−0.058^***^	0.036^***^	0.029^***^	−0.102^***^	0.048^***^	0.033^***^	−0.035^***^	0.036^***^
	(0.004)	(0.003)	(0.004)	(0.004)	(0.009)	(0.004)	(0.004)	(0.010)	(0.004)
age2	−0.015^***^	0.036^***^	−0.019^***^	−0.021^***^	0.063^***^	−0.033^***^	−0.016^***^	0.036^***^	−0.020^***^
	(0.004)	(0.003)	(0.004)	(0.004)	(0.008)	(0.004)	(0.004)	(0.010)	(0.004)
marriage	−0.053^*^	0.104^***^	−0.065^**^	−0.010	0.223^***^	−0.051^*^	0.008	0.577^***^	−0.047
	(0.030)	(0.022)	(0.030)	(0.032)	(0.061)	(0.030)	(0.031)	(0.073)	(0.030)
child	−0.236^***^	0.161^***^	−0.254^***^	−0.532^***^	−1.053^***^	−0.337^***^	−0.395^***^	−0.331^***^	−0.364^***^
	(0.045)	(0.032)	(0.045)	(0.046)	(0.090)	(0.043)	(0.045)	(0.108)	(0.044)
job1	0.618^***^	−0.200^***^	0.641^***^	0.891^***^	1.343^***^	0.642^***^	0.654^***^	0.811^***^	0.578^***^
	(0.027)	(0.019)	(0.027)	(0.027)	(0.053)	(0.026)	(0.027)	(0.065)	(0.027)
job3	0.407^***^	0.013	0.405^***^	0.939^***^	2.417^***^	0.490^***^	0.590^***^	2.172^***^	0.385^***^
	(0.031)	(0.022)	(0.031)	(0.030)	(0.058)	(0.029)	(0.031)	(0.073)	(0.031)
health			0.115^***^	0.147^***^	0.274^***^	0.096^***^	0.140^***^	0.377^***^	0.105^***^
			(0.010)	(0.010)	(0.020)	(0.010)	(0.010)	(0.024)	(0.010)
_cons	−0.001	5.007^***^	−0.578^***^	0.172	26.909^***^	−4.820^***^	−0.164	8.820^***^	−0.997^***^
	(0.095)	(0.068)	(0.107)	(0.112)	(0.216)	(0.139)	(0.108)	(0.258)	(0.109)
Indirect effect			0.003			0.127			0.097
95 CI			[0.002, 0.005]			[0.115, 0.140]			[0.090, 0.104]

^*^, ^**^ and ^***^ represent 10%, 5%, and 1% significance levels.

### 3.5 Analysis of changes over time

Table 7 presents the regression results of the disparities in physical activity levels between urban and rural residents. Here, *N* denotes the sample size, and *R*^2^ indicates the goodness of fit. Columns (1) and (2) display the regression outcomes for urban and rural samples in 2010, while columns (3) and (4) show the results for urban and rural samples in 2021. The subsequent analysis examines the variations in key variables over time.

(1) Income level: In 2010, an association has been found between income level and physical activity in both urban and rural areas, with respective *p*-values of 0.027 and 0.01, respectively, although the effect was stronger in urban area. By 2021, the effect increased in both, with a more pronounced increase in rural area at the significance level of 0.039, suggesting that income plays an increasingly important role in physical activity in rural settings.
(2) Years of education: In 2010, education had a positive impact in both urban and rural areas at the coefficients of were 0.087 and 0.067, respectively, with a stronger effect in urban areas. By 2021, the effect remained strong in urban areas and increased substantially in rural areas at the coefficients of 0.088 and 0.136, respectively, There is an increasing link between education and physical activity in rural communities.
(3) Social interaction frequency: In 2010, social interaction had a positive role in urban areas than in rural areas, with the coefficients of 0.218 and 0.135, respectively. By 2021, the effect decreased in urban areas, but increased in rural areas, with the coefficients of 0.088 and 0.153, possibly reflecting changes in the nature and content of social activities in rural areas.
(4) Internet use frequency: In 2010, internet use had a larger positive effect in rural areas than in urban areas, with the coefficients of 0.194 and 0.092, respectively. By 2021, the pattern reversed: the effect increased in urban areas, but decreased in rural areas, with the coefficients of 0.117 and 0.047, respectively, which may be related to differences in internet penetration and usage patterns.

**Table 7:** Regression results by urban/rural areas and year.

Variable	2010		2021	
	(1) Urban	(2) Rural	(3) Urban	(4) Rural
Lninc	0.027^***^	0.010^**^	0.032^***^	0.039^***^
	(0.004)	(0.004)	(0.006)	(0.006)
Edu	0.087^***^	0.067^***^	0.088^***^	0.136^***^
	(0.005)	(0.005)	(0.006)	(0.008)
Social	0.218^***^	0.135^***^	0.088^***^	0.153^***^
	(0.017)	(0.015)	(0.020)	(0.022)
net	0.092^***^	0.194^***^	0.117^***^	0.047^***^
	(0.013)	(0.019)	(0.017)	(0.018)
Gender	0.103^***^	−0.023	0.079^*^	0.006
	(0.034)	(0.031)	(0.046)	(0.053)
Age	0.046^***^	0.011	0.047^***^	0.019^*^
	(0.007)	(0.007)	(0.009)	(0.011)
age2	−0.019^***^	−0.002	−0.031^***^	−0.009
	(0.007)	(0.006)	(0.008)	(0.010)
Marriage	−0.020	−0.068	−0.024	−0.014
	(0.051)	(0.050)	(0.061)	(0.070)
Child	−0.253^***^	−0.212^***^	−0.099	−0.239^*^
	(0.070)	(0.075)	(0.091)	(0.122)
Health	0.115^***^	0.021	0.115^***^	0.059^**^
	(0.017)	(0.014)	(0.024)	(0.023)
job1	0.567^***^	0.147^***^	0.472^***^	0.193^**^
	(0.048)	(0.038)	(0.082)	(0.090)
job3	0.194^***^	0.116^*^	0.077	0.061
	(0.048)	(0.060)	(0.081)	(0.114)
_cons	−1.657^***^	0.083	−0.877^***^	−0.331
	(0.187)	(0.180)	(0.258)	(0.314)
*N*	7,097	4,520	4,542	3,544
*R* ^2^	0.194	0.147	0.109	0.161

^*^, ^**^ and ^***^ represent 10%, 5%, and 1% significance levels.

## 4 Discussion

### 4.1 Urban–rural disparities in physical activity and their temporal changes

Empirical analysis indicates that between 2010 and 2021, the disparity in physical activity levels between urban and rural residents exhibited the following characteristics: overall levels increased, while the gap narrowed but remained significant. This trend is closely linked to China’s socioeconomic development and the effects of policy interventions (20). In 2010, the average frequency of physical exercise among urban residents was 1.71 times that of rural residents; by 2021, this ratio had decreased to 1.31 times, reflecting the positive outcomes of policies aimed at achieving equity in physical activity between urban and rural areas. From a demographic perspective, the characteristics of the rural sample—including a higher average age, a higher proportion of manual laborers and fewer years of education—reflect patterns of urban–rural disparities and factors associated with physical activity participation. These factors—including declining physical function among the old adults, high levels of work-related fatigue among manual laborers, and limited health awareness among those with lower education levels (21)—are associated with the persistent trend of lower exercise frequency among rural residents compared to urban residents. The uniqueness of this study lies in the following: first, it utilizes data from the CGSS surveys conducted in 2010 and 2021, which span over a decade and employ consistent measurement methods. Second, by employing model specifications and mediation analysis to control for differences between urban and rural residents in terms of economic income, educational attainment, social interaction, internet usage, and personal characteristics, the results still indicate that the disparity in physical exercise participation between urban and rural residents has significantly narrowed over the past decade of development in the new era. This finding fully demonstrates the effectiveness of China’s policies aimed at promoting sports equity between urban and rural areas. The continued implementation of the National Fitness Program (2011–2015) and the National Fitness Program (2016–2020), coupled with the vigorous advancement of the strategy to build a sports powerhouse and the Healthy China initiative, has enabled urban and rural sports to gradually enter a new phase of coordinated and integrated development.

### 4.2 Intrinsic mechanisms and changes in urban–rural physical activity

The intrinsic mechanisms underlying urban–rural disparities in physical activity are multidimensional, complex, and dynamic. Through regression analysis of factors such as income level, years of education, social interaction, and internet use, this study systematically uncovers these mechanisms and their evolution over time.

(1) Income level is a significant factor associated with, or potentially an explanatory pathway for, differences in the frequency of physical activity among urban and rural residents. Higher-income families are better able to afford expenses related to physical activity, such as sports equipment, venue rentals, and professional instruction (22). Both the 2010 and 2021 data show a positive correlation between income and physical activity in urban and rural areas, with a more pronounced effect in rural areas in 2021. As the income level of rural residents gradually rises, their interest in healthy lifestyles grows (15), Income level is a significant factor associated with, or potentially an explanatory pathway for, differences in the frequency of physical activity among urban and rural residents.
(2) Years of education is a factor associated with differences in the frequency of physical activity, and this association is significant. Individuals with higher educational attainment typically possess stronger health awareness and exercise conscientiousness. In 2010, education had a notable positive impact on physical activity for both urban and rural residents, with a stronger effect in urban areas. By 2021, this impact became even more pronounced in rural areas, suggesting that as educational attainment in rural areas improves, the role of education in promoting physical activity will become increasingly important.
(3) The relationship between the frequency of social interaction and physical activity varies across different time periods and regions. In 2010, urban residents engaged in more frequent social interactions, which in turn supported higher physical activity frequency. By 2021, this effect weakened in urban areas but strengthened in rural areas. This may reflect changes in the forms and content of socialization in rural areas (23), with more group activities promoting physical activity.
(4) Internet use provides residents with new ways to access information and resources related to physical activity and exhibits different trends over time and across regions. In 2010, there was a significant correlation between internet use and physical activity among rural residents. By 2021, this effect increased in urban areas, but weakened in rural areas. This shift may be associated with changes in internet penetration and usage patterns in urban areas. With the proliferation of smartphones and internet access in rural areas, the impact of the internet on physical activity is beginning to emerge (24), although it is not yet as extensive and profound as in urban areas.

### 4.3 International comparison of research findings and their distinctiveness in a global context

This study found that the frequency of physical exercise among both urban and rural residents in China showed a significant upward trend from 2010 to 2021, with the urban–rural gap gradually narrowing. In contrast, India—a developing country comparable to China in terms of population size and development stage—conducted a nationwide longitudinal study revealing no significant improvement in overall physical activity levels among residents between 2010 and 2020 (25); in fact, a slight decline was observed in rural areas. Although the Indian government implemented similar public health initiatives during this period, such as the “National Health Policy,” aimed at improving residents’ health behaviors, these policies failed to translate into substantial increases in physical activity participation rates. Contributing factors included the uneven distribution of medical resources between urban and rural areas, insufficient health education coverage in rural regions, and slow progress in improving residents’ health literacy. Furthermore, panel data analyses of developing countries in Latin America, such as Brazil and Mexico, have shown that as urbanization accelerates, residents’ leisure activities are increasingly shifting toward sedentary behaviors (26). Despite increased government investment in sports facility construction, physical activity levels have remained stagnant; in some low-income groups, physical activity has even declined due to rising employment pressures and reduced time available for exercise. Turkey also faces the challenge of declining health due to reduced physical activity. Two cross-sectional studies conducted among adults aged 25 to 64 in Türkiye found that, despite societal development, there were no significant changes in physical activity levels or obesity prevalence between 2014 and 2023. Although the Ministry of Health implemented various initiatives during this period to promote physical activity, their overall impact was minimal (27). Similar findings emerged from a global survey of physical activity levels across 168 countries. Between 2001 and 2016, no significant increase in physical activity was observed in countries across multiple regions, including Southeast Asia, Oceania, and Africa (28). However, the prevalence of physical inactivity was significantly higher in high-income countries compared to low-income countries over time.

Compared to the aforementioned countries, China’s uniqueness is evident in three core aspects. First, the systematic nature of top-level design: the “Healthy China” strategy elevates national fitness to a top priority. Through a series of policy documents, such as the “National Fitness Plan,” it has established a multifaceted promotion mechanism characterized by “government leadership, social participation, and market operation,” ensuring the continuity and targeted nature of policy interventions. Second, the implementation of integrated urban–rural development: by addressing the shortage of sports facilities in rural areas with targeted measures such as “universal coverage of village-level fitness squares” and “sending sports instructors to rural areas,” China effectively narrows the gap in sports resources between urban and rural areas, providing a foundational guarantee for rural residents’ participation in physical activities. Third, the breadth of social mobilization, which leverages multiple channels—including media campaigns, community activities, and school sports—to deeply embed the health concept of “active exercise” into the lives of urban and rural residents, gradually fostering a sports culture of universal participation. These characteristics collectively have driven the sustained rise in physical activity levels in China. They also demonstrate that, in the context of developing countries, relying solely on economic development or spontaneous shifts in health behaviors is insufficient to achieve a nationwide increase in physical activity; rather, systematic policy interventions, balanced resource allocation, and broad-based social mobilization are the key driving factors.

## 5 Conclusion

The findings of this study can be summarized as follows:

(1) Increased but unequal physical activity: Between 2010 and 2021, the frequency of physical activity increased for both urban and rural residents. However, urban residents consistently reported significantly higher exercise frequency than rural residents, indicating the imbalances in sports resources and economic conditions between urban and rural areas. (2) A variety of factors are closely linked to differences in the frequency of physical exercise. Factors such as income level, years of education, social interaction, and internet usage all show a significant correlation with the frequency of physical exercise. (3) Long-term observations indicate that policy effectiveness remains insufficient. Although the National Fitness Program and the Healthy China Initiative are significantly associated with residents’ physical activity levels, disparities between urban and rural areas persist. Rural areas lag behind in terms of economic development, educational attainment, and internet penetration, and these factors are closely linked to differences in the frequency of physical exercise.

In order to further narrow the urban–rural gap in physical activity and promote the comprehensive implementation of the national fitness and healthy China strategies, the following policy recommendations are proposed: (1) Increase investment in rural exercise infrastructure: The government should enhance investment in sports facilities in rural areas to ensure that rural residents have convenient access to high-quality venues and equipment. Simultaneously, social capital should be encouraged to participate in the development of rural exercise infrastructure to establish a diversified investment mechanism. (2) Targeted interventions in rural areas: Policies should prioritize industrial development and employment opportunities in rural areas to boost farmers’ income, and improve their ability to participate in sports and exercise. Education departments should design specific programs to disseminate scientific exercise knowledge and enhance the health literacy of rural residents. Community sports activities should be organized to increase socialization opportunities and stimulate interest and motivation for physical activity. Additionally, improving internet penetration in rural areas can help residents access more exercise guidance and support. (3) Develop tailored public health policies: When formulating public health policies, the government should fully consider urban–rural disparities and implement tailored health promotion strategies. Policies targeting rural areas should be precise and address practical issues to enhance their relevance and effectiveness.

## 6 Limitations

This study provides valuable insights into revealing the differences in physical exercise between urban and rural areas and their underlying mechanisms. However, its conclusions need to be interpreted cautiously in the context of the following limitations. Firstly, the core limitation stems from the nature of cross-sectional data, which means that the associations revealed (such as the positive correlation between factors like income and education and exercise frequency) cannot be established as causal effects. Reverse causality (e.g., exercise behavior enhancing health awareness) and omitted variables that are difficult to measure (such as personal endowments and community culture) may lead to estimation bias. Secondly, the measurement of physical exercise relies on self-reported frequency, which may be influenced by recall bias, and fails to capture dimensions such as intensity, duration, and specific forms. The absence of objective indicators (such as metabolic equivalent) also limits the application value of the results in precise health guidance. Furthermore, despite controlling for multiple types of variables, psychological factors, community built environment, and deeper cultural backgrounds were not included in the model, which may affect the completeness of the mechanism analysis. Lastly, although data from 2010 and 2021 were compared to observe trends, it is difficult to accurately depict the dynamic trajectory and nonlinear characteristics of the evolution of differences based on only two time points. In the future, more time points should be included for longitudinal tracking analysis.

## Data Availability

The original contributions presented in the study are included in the article/supplementary material, further inquiries can be directed to the corresponding author.
